# Medial Parabrachial Nucleus Is Essential in Controlling Wakefulness in Rats

**DOI:** 10.3389/fnins.2021.645877

**Published:** 2021-03-25

**Authors:** Qi Xu, Dian-Ru Wang, Hui Dong, Li Chen, Jun Lu, Michael Lazarus, Yoan Cherasse, Gui-Hai Chen, Wei-Min Qu, Zhi-Li Huang

**Affiliations:** ^1^Department of Physiology, School of Basic Medical Sciences, Anhui Medical University, Hefei, China; ^2^Department of Pharmacology, School of Basic Medical Sciences, Fudan University, Shanghai, China; ^3^State Key Laboratory of Medical Neurobiology, MOE Frontiers Center for Brain Science, Institutes of Brain Science, Fudan University, Shanghai, China; ^4^Department of Neurology, Beth Israel Deaconess Medical Center, Harvard Medical School, Boston, MA, United States; ^5^International Institute for Integrative Sleep Medicine (WPI-IIIS), University of Tsukuba, Tsukuba, Japan; ^6^Department of Sleep Disorders and Neurology, The Affiliated Chaohu Hospital of Anhui Medical University, Hefei, China

**Keywords:** chemogenetics, glutamatergic neurons, optogenetics, parabrachial nucleus, rat, wakefulness

## Abstract

Activation of the parabrachial nucleus (PB) in the brainstem induced wakefulness in rats, suggesting which is an important nucleus that controls arousal. However, the sub-regions of PB in regulating sleep-wake cycle is still unclear. Here, we employ chemogenetics and optogenetics strategies and find that activation of the medial part of PB (MPB), but not the lateral part, induces continuous wakefulness for 10 h without sleep rebound in neither sleep amount nor the power spectra. Optogenetic activation of glutamatergic MPB neurons in sleeping rats immediately wake rats mediated by the basal forebrain (BF) and lateral hypothalamus (LH), but not the ventral medial thalamus. Most importantly, chemogenetic inhibition of PB neurons decreases wakefulness for 10 h. Conclusively, these findings indicate that the glutamatergic MPB neurons are essential in controlling wakefulness, and that MPB-BF and MPB-LH pathways are the major neuronal circuits.

## Introduction

Wakefulness has been reported to be controlled by multiple neuronal systems, such as histamine neurons in the tuberomammillary nucleus (TMN) (Huang et al., 2006), noradrenaline (NA) neurons in the locus coeruleus (LC) (Hagan et al., 1999), orexinergic neurons in the lateral hypothalamus (LH) (Chemelli et al., 1999), and GABAergic and cholinergic neurons in the basal forebrain (BF) (Anaclet et al., 2015; Xu et al., 2015). However, lesions or inactivation of single arousal system demonstrated that none of these arousal nuclei are key players in initiating or maintaining wakefulness (Blanco-Centurion et al., 2007), which indicates that wakefulness may be regulated by arousal promoting networks, or that more essential nuclei controlling wakefulness remain unidentified.

In humans, brainstem stroke patients often experience coma symptoms when brain damage is confined in the upper pontine tegmentum (Parvizi and Damasio, 2003). In rats, bilateral chemical lesions of the parabrachial nucleus (PB) area, including the precoeruleus, induce a deep coma state with behavioral unresponsiveness (Fuller et al., 2011). Recently, Qiu et al. (2016) showed that chemogenetic activation of the PB induced long lasting arousal via the BF and LH in rats; however, the wakefulness induced by activation of PB may be secondary to other behavior or physiologic functions (Scammell et al., 2017). The effect of reversible inhibition of the PB on wakefulness regulation is needed. In addition, specific lesions of glutamatergic neurons in the external and crescent parts of lateral PB (LPB) but not the medial part of PB (MPB) decreased hypercapnia-evoked arousal, while specific deletion of glutamatergic MPB neurons increased non-rapid eye movement (NREM) sleep in mice (Kaur et al., 2013). Moreover, many of the LPB neurons that express calcitonin gene-related peptide are responding to CO_2_, and activation of these neurons in the LPB induced wakefulness, while inhibition of these neurons prevented arousal to CO_2_ in mice (Kaur et al., 2017). In addition, electrical activation of the LPB can induce reanimation during continuous isoflurane anesthesia (Muindi et al., 2016), and chemogenetic activation of glutamatergic PB neurons shorten the anesthesia recovery time in mice (Wang et al., 2019; Xu et al., 2020). These results indicated that the PB may be a powerful candidate in sleep-wake regulation, and the MPB and LPB may play different roles. Moreover, the sub-regions of the PB that are involved in natural wakefulness and the cellular types of the downstream targets are still unclear.

In the current study, we employ a chemogenetic strategy to activate PB neurons, and elucidate the role of MPB and LPB in promoting wakefulness. In addition, optogenetic strategy is employed to activate the glutamatergic MPB neurons and their terminals in the BF, LH and ventral medial thalamus (VM), to reveal which cell types of BF, LH, or VM neurons, are responsible for PB in controlling arousal. Lastly, inhibition of PB by genetically engineered ivermectin (IVM)-gated human glycine receptor (IVMR) was employed to decrease wakefulness in rats. These results will clearly reveal subregions of the PB and their neural circuits in controlling wakefulness.

## Materials and Methods

### Animals

Pathogen-free adult Sprague–Dawley rats (male, weighing 45–55 g, 3 weeks old, or 200–220 g, 6 weeks old) were purchased from the Sino-British SIPPR/BK Lab. Animal LTD., Shanghai, China. The rats were housed at an ambient room (temperature, 23 ± 1°C and relative humidity, 60 ± 5%) under automatically controlled 12 h/12 h light/dark cycle condition (07:00/19:00). The animals had access the water and food *ad libitum* during the study (Zhang et al., 2017; Dong et al., 2019). All experiments were carried out in accordance with the National Institutes of Health Guide for the Care and Use of Laboratory Animals and approved by the Animal Care and Use Committee of Fudan University. No sample size calculation was performed. The sample size used in present study is depended on the expected variations between rats and is comparable to many previous reports using similar techniques. Additionally, no method of randomization or blinding of treatment was used in present study.

### Generation of Adeno-Associated Viral (AAV) Vectors

The AAVs of serotype rh10 for AAV-hSyn- hM3Dq-mCherry, AAV-hSyn-mCherry were generated by tripartite transfection into 293A cells, separately, as we described previously (Lazarus et al., 2011; Oishi et al., 2017). The AAV-hSyn-IVMR-eGFP and AAV-hSyn-eGFP were purchased from Shanghai Taiting biological Co. Ltd. (Shanghai, China). The AAV-CaMKIIα-ChR2-mCherry and AAV-CaMKIIα-mCherry were purchased from Obio Technology Co. Ltd. (Shanghai, China).

### Stereotaxic AAV Injection and Electrode Implants

The animals were anesthetized with chloral hydrate (10% in saline, 350 mg/kg), using aseptic techniques, hM3Dq or mCherry (200 nL/injection) was injected stereotaxically into the MPB (AP = −8.0 mm, ML = ± 1.5 mm, DV = −5.8 mm), or LPB (AP = −8.0 mm, ML = ± 2.2 mm, DV = −5.0 mm) according to the rat brain atlas of Paxinos and Watson (2007) in 6-week-old rats. Another batch of rats were microinjected with IVMR or eGFP (200 nL/injection) into the PB (AP = −8.0 mm, ML = ± 1.7 mm, DV = −5.8 mm) bilaterally. Then, the rats were implanted the EEG and EMG electrodes as described before (Zhang et al., 2013; Chen et al., 2019; Li et al., 2020; Shen et al., 2020). For optogenetics, ChR2 or mCherry (200 nL/injection) was stereotaxically injected into the MPB region, and 2 weeks later, the EEG/EMG electrodes and guide cannula for optic fibers were implanted. Following surgery, rats were housed individually for 2 weeks (Wu et al., 2015; Oishi et al., 2017; Luo et al., 2018; An et al., 2020).

### EEG/EMG Recording and Sleep-Wake Scoring

Rats were allowed 14 days recovery from surgery before the EEG/EMG recording. Each rat was connected to an cable for EEG/EMG recording in a chamber and habituated for 3 days before the recording. The EEG/EMG signals (EEG: 0.5–30 Hz, EMG: 20–200 Hz, sampling rate: 128 Hz) were recorded at baseline and under chemogenetic or optogenetic manipulation conditions using Vitalrecorder software (Kissei Comtec, Nagano, Japan). Then the vigilance states were automatically scored offline by 10 s epochs into three stages, including wake, REM or NREM sleep, using Sleepsign (Kissei Comtec, Nagano, Japan), according to previously established criteria (Xu et al., 2014; Li et al., 2020; Shi et al., 2020). As a final step, defined sleep-wake stages were checked visually, and corrected if necessary. The amount of time spent in each vigilance stage was determined from the scored data. The EEG power spectral density was converted into a dataset in 10-s epochs for 0–25 Hz, 24 h in length of sleep-wake behavior in the chemogenetics data or 3 h in length in the optogenetics data. The bit map represents the EEG power spectra generated by MATLAB (The MathWorks, Inc., Massachusetts) (Litvak et al., 2011).

### Drug Treatments

Clozapine-*N*-Oxide (CNO) was purchased from LKT Laboratories, Inc. (Saint Paul, MN, United States). The CNO (0.03, 0.1, or 0.3 mg/kg) was dissolved in saline and intraperitoneally injected in rats at 09:00 for hM3Dq rats. The IVM were purchased from Sigma-Aldrich (Missouri), and dissolved in isopropanol at the dosage of 10 mg/kg, The IVM and isopropanol were administrated at 20:00 on a 2-day schedule.

### Behavioral Analysis

The behaviors of the rats after saline or CNO injection were analyzed using video recordings as we described previously (Oishi et al., 2017). Briefly, behaviors during the first hour and the third hour after saline or CNO treatment were scored in 4 s epochs as attentive wake, characterized by non-specific motor activity (for example, head bobbing and low neck muscle activity.) or quiet wake, during which animals were quiet without walking (Lepski et al., 2012), and grooming (including head washing, body grooming, and paw or leg licking), exploring, eating, drinking and sleep when the behavior accounted for more than 50% of the epoch.

### *In vivo* Optogenetics

Fourteen days after the surgery for implanting the EEG/EMG electrodes and optic guide cannula, the EEG/EMG recording cables were connected to the amplifier and fiber optic cables (1-m long, 200-μm diameter, Newdoon Inc., Hangzhou, China) were placed inside the implanted cannula simultaneously, and fiber-optic rotary joints (Doric Lenses, Québec, Canada) were used for unrestricted *in vivo* illumination. Rats were acclimatized for 2 days before the photostimulation sessions. Light pulse trains were programmed using a pulse generator (Nihon Kohden, Tokyo, Japan) that provided simultaneous input into 2 blue light lasers (473 nm, 100 mW intensity; SLOC, Shanghai, China). For acute optogenetic procedure, each stimulation train was applied 60 s after a stable NREM or REM sleep event as detected by real-time online polysomnographic recording. For the chronic photostimulation experiments, blue light stimulation (5-ms pulses, 50 trains of 20 Hz for MPB, 25 trains of 40 Hz for BF, LH and VM, main interval 30 s) was applied for 1 h during 9:00-10:00.

### *In vitro* Electrophysiology

For *in vitro* electrophysiologic recording, 3-week-old rats were injected with recombinant AAVs carrying ChR2 or mCherry (200 nL/injection) for the optogenetics experiment into the MPB (AP = −7.0 mm, ML = ± 1.6 mm, DV = −5.8 mm). After 3–4 weeks of postoperative recovery, rats were anesthetized and perfused transcardially with ice-cold modified artificial cerebrospinal fluid (aCSF) containing (in mM) 0.4 Vitamin C, 0.5 CaCl_2_, 1.2 NaH2PO_4_, 2 Na-pyruvate, 2.5 KCl, 3 MgSO_4_, 10 glucose, 23 NaHCO_3_, 252 sucrose, and saturated with 95% O_2_ and 5% CO_2_ (pH 7.2–7.4, 301–305 Osm). Coronal slices (250 mm thick) containing the PB were cut using a vibratome (VT1200S, Leica, Germany) and incubated for 1 h at 32°C in a holding chamber in oxygenated aCSF containing (in mM) 1.25 NaH2PO_4_,1.3 MgSO_4_, 2 CaCl_2_, 3 KCl, 10 glucose, 26 NaHCO_3_, and 124 NaCl.

Whole-cell recordings were performed using patch electrodes (4–6 MΩ) containing (in mM) 0.3 EGTA, 0.3 Na-GTP, 4 Mg-ATP, 10 KCl, 10 Na-phosphocreatine, 10 HEPES, 125 potassium gluconate, and 0.2% biocytin (PH 7.3; Osmolarity, 290 ∼ 300 mOsm). The slice was transferred to a recording chamber which was continuously perfused with oxygenated aCSF at a flow rate of 2–3 ml/min (32°C). The PB was identified by its localization relative to the superior cerebellar peduncle and fourth ventricle under visual guidance using a fluorescence microscope (Olympus, Tokyo, Japan). Recorded PB neurons were further distinguished from other cells by positive fluorescence. Coronal sections of the PB, BF, LH, or VM (300 μm) were collected. ChR2 was stimulated by a 473 nm blue light laser (SLOC, Shanghai, China). The tip of the optical fiber was placed 500 μm above the recording cell. Cells with series resistance changed by > 20% were discarded.

### Single-Cell Reverse Transcription (RT-PCR)

After each recording, cytoplasm was aspirated into the patch pipette by applying negative pressure, and expelled into a PCR tube (Axygen, Massachusetts) as previously described (Xu et al., 2015). The presence of mRNAs coding for ChAT, VGluT2, and VGAT was detected by single cell RT-PCR, according to the manufacturer’s instructions (Supplementary Table 1). Then, PCR products were visualized by Safe Gel-stained 1.5% agarose gel electrophoresis.

### Immunohistochemistry

Animals were deeply anesthetized with chloral hydrate (400 mg/kg, i.p.) and perfused transcardially with saline followed by 4% paraformaldehyde. Brain samples were removed and postfixed in 4% paraformaldehyde overnight at 4°C, and cryoprotected in 20% sucrose-phosphate buffer (4°C) until sunk to the bottom. The brain samples were then frozen and sectioned in the coronal plane at 30 μm using a Leica freezing cryotome (CM1520, Leica, Germany). The staining was performed on free-floating sections as previously described (Zhang et al., 2013; Xu et al., 2019). In brief, sections were incubated with the primary antisera (rabbit anti c-Fos, 1:10000, Millipore, Massachusetts). at room temperature overnight. Then the sections were rinsed by PBS and incubated for 1.5 h in biotinylated anti-rabbit secondary antiserum (1:1000, Jackson Immunoresearch Laboratories, Pennsylvania). All tissue sections were manipulated with avidin-biotin complex (1:1000, Vector Laboratories, California) for 1 h, and immunopositive cells were visualized black by reaction with 3,3-diaminobenzidine (DAB) with nickel (DAB Substrate Kit, Vector Laboratories, California). After rinsing with PBS, the sections were once more incubated with the primary antibody for anti-Dsred (1:5000, Takara Bio Inc., Shiga, Japan). Following this incubation with secondary antibody and avidin-biotin complex, the sections were visualized brown by reaction with DAB without nickel. Following additional washes in PBS, sections were then mounted, dried, dehydrated, and cover slipped. The hM3Dq or ChR2 expression was identified by the expression of mCherry positive neurons by either only staining the DsRed/mCherry, or viewing the native fluorescence of PB by microscope (Olympus, BX51, Tokyo, Japan) with a boundary that encompassed >90% of all mCherry-containing neurons. IVMR expression was identified by staining against eGFP (1:1000, life technologies, California). The c-Fos positive neurons were counted to evaluate the potential neuronal circuits mediating the wake-promoting effect of MPB, including MPB, CG, M1, S1, LS, BNST, CeA, BF, MDM, LPMR, PV, VM, LH, PSTN, TMN, MGV, PAG, VTA, and LC. Cell counting was performed on three adjacent sections (separated by 90 μm) from four rats, the average counting per section per side was used to represent the data.

According to the rat brain atlas in stereotaxic coordinates by Paxinos and Watson (2007), the boundaries of PB was defined along the superior cerebellar peduncle. For all the rats were included in the analysis only if above 80% of the labeled neurons were central in the target region (MPB or LPB, respectively) bilaterally with a small transfection of adjacent area. Of the rats for analysis from the optogenetic study, 7 cases of mCherry expression missed the MPB bilaterally were excluded, and 3 missed the BF bilaterally and 2 missed the LH bilaterally were excluded. In total, 12 cases of rats were excluded from the optogenetic study *in vivo*.

### Statistical Analysis

All results were expressed as the mean ± SEM. Statistical analysis between 2 groups was performed using the paired or unpaired two-sided Student’s *t*-test. In all cases, *P* < 0.05 were taken as the level of significance.

## Results

### MPB but Not LPB Neurons Were Involved in Controlling Wakefulness in Rats

**FIGURE 1:**
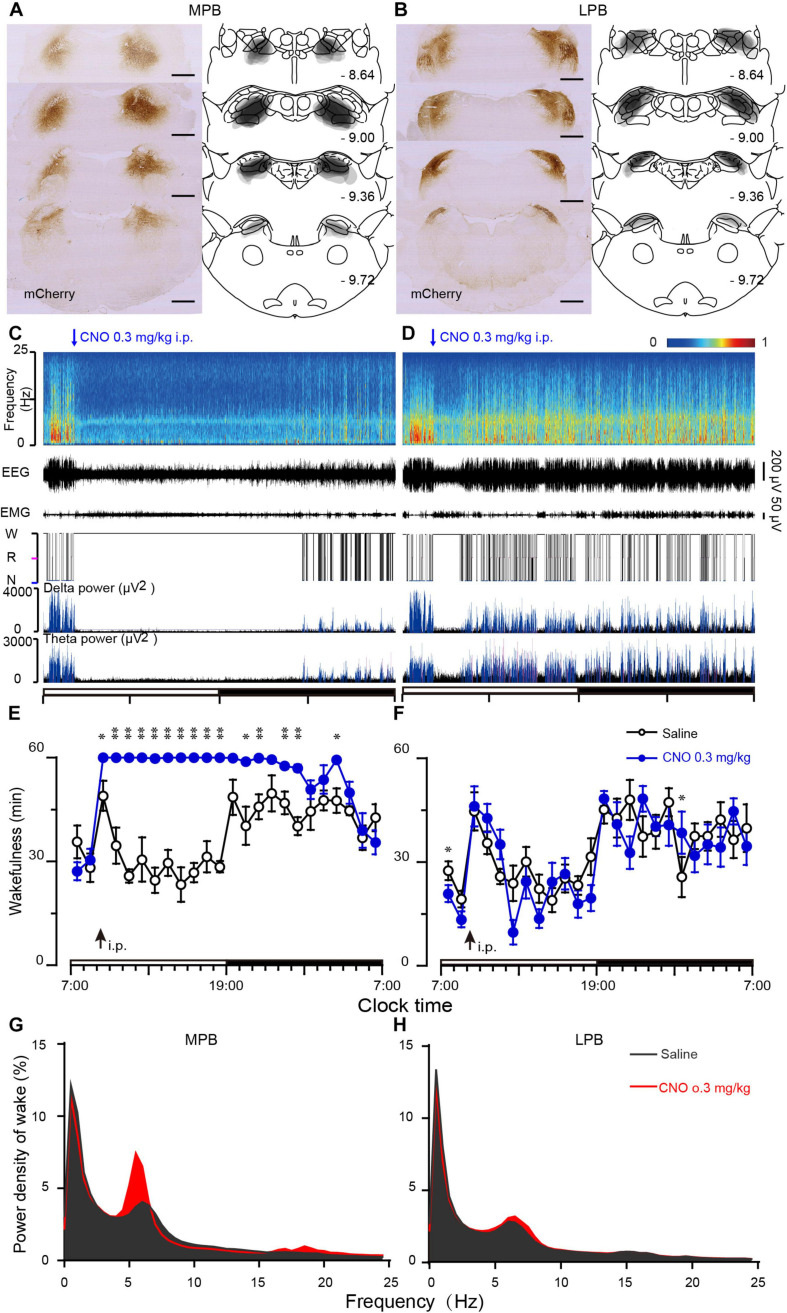
Chemogenetic activation of MPB (but not LPB) neurons induces wakefulness. **(A,B)** Schematic diagram indicating the position of hM3Dq-mCherry protein was confined to the MPB **(A)** or LPB **(B)**. Superimposed mCherry expression areas in the MPB **(A)** and LPB **(B)** of 8 AAV-injected rats in each group, are shown in the right panels (Paxinos and Watson, 2007). Scale bars: 1 mm. **(C,D)** Typical examples of EEG power spectra, EEG, EMG, hypnogram, and relative magnitude changes in delta and theta power over 24 h for a rat expressing hM3Dq at MPB **(C)** or LPB neurons **(D)** when given CNO (0.3 mg/kg). **(E,F)** Time courses of wakefulness after administration of CNO (0.3 mg/kg) or saline in rats expressing hM3Dq in MPB **(E)** or LPB neurons **(F)**. The arrow indicates the time point of CNO or saline administration, and open and closed bars above the *x*-axis indicate light and dark periods, respectively. **(G,H)** EEG Power spectrum of wakefulness during 10 h (9:00–19:00) after the CNO injection in MPB-hM3Dq **(G)** and LPB-hM3Dq rats **(H)**. *P < 0.05, **P < 0.01, compared with saline (*n* = 8).

**FIGURE 2:**
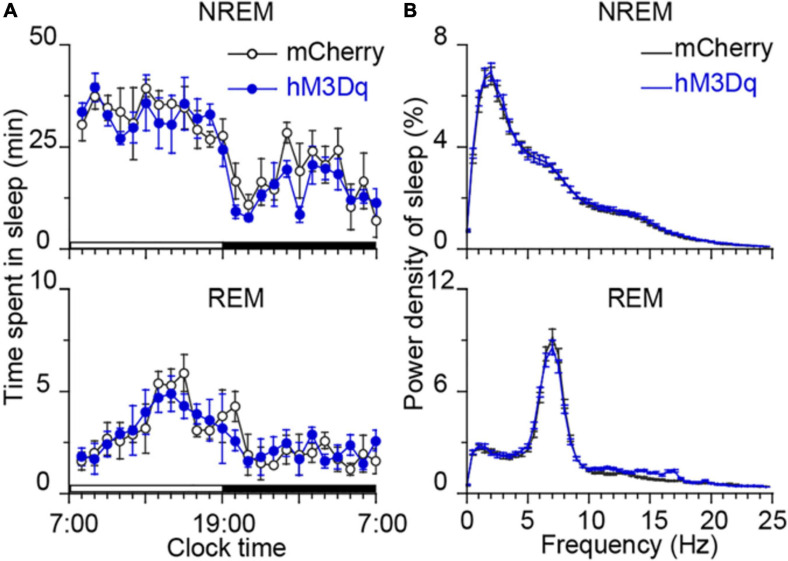
Time course of sleep and power spectra starting 22 h after CNO treatment. **(A)** No bouts of NREM and REM sleep after the CNO treatment. Each circle represents the hourly mean amount of sleep. The horizontal open and closed bars on the *x*-axes indicate the 12-h light and 12-h dark periods, respectively. **(B)** EEG power spectra of NREM and REM sleep during the following day in rats injected with AAV-hSyn-hM3Dq-mCherry in MPB (*n* = 8).

To determine the sub-regions of the PB neurons in controlling wakefulness, the AAV vector containing the excitatory mutant human M3 muscarinic receptors (hSyn-hM3Dq-mCherry-AAV, hM3Dq) were expressed in bilateral parts of either the MPB or LPB. As shown in Figures 1A,B, the hM3Dq/mCherry fusion protein was successfully expressed in the MPB (Figure 1A) or LPB (Figure 1B), as indicated by superimposed mCherry expression areas in 8 AAV-injected rats, respectively. Administration of CNO (0.3 mg/kg) promoted long-lasting wakefulness in rats expressing hM3Dq in the MPB (Figures 1C,E) without rebound in sleep or changes in the power spectrum for NREM and REM sleep after long-lasting wakefulness induced by CNO (Figure 2), similar to activation of the entire PB (Qiu et al., 2016). In contrast, there was no significant change in the sleep-wake profiles of rats after activation of only the LPB (Figures 1D,F). In addition, the CNO treated MPB-hM3Dq rats showed an increased theta EEG power spectra of wakefulness compared with the saline-treatment (Figure 1G), while the CNO-treatment did not change the EEG power spectrum of wakefulness in the LPB-hM3Dq rats (Figure 1H). These data clearly indicate that MPB neurons play a crucial role in controlling wakefulness.

**FIGURE 3:**
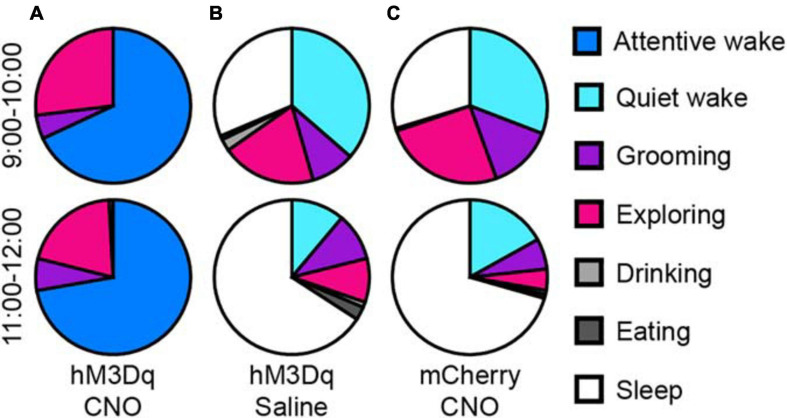
Activation of glutamatergic PB neurons produces attentive wake in rats. After injection of CNO at 9:00, rats are mainly in attentive wake (do not move but with head bobbing) with modest amounts of time spent exploring, grooming, or rearing for more than 10 h **(A)**. After injection of saline, hM3Dq rats are mainly asleep with a small amount of time in quiet wake and exploring behavior **(B)**. Injections of CNO in mCherry control rats at 9:00 produced similar behavior to the saline treated hM3Dq rats, mainly asleep with a small amount of time in quiet wake and exploring, grooming, and feeding **(C)** (*n* = 4).

We analyzed the behaviors of rats during long-lasting arousal induced by CNO. During 10 h after CNO administration (light period), rats spent most of their time in “attentive wake” characterized by head bobbing without moving around, while these rats after saline treatment spent more time in “quiet wake” and sleep during the first hour, followed by more sleep during the subsequent periods (Figure 3 and Supplementary Movies 1, 2). After saline treatment, the rats showed exploratory behaviors, such as exploring, sniffing and rearing during the first hour (Figure 3B and Supplementary Movie 2). The mCherry-expressing control rats treated with CNO at 9:00 also exhibited a similar behavior as the hM3Dq rats treated with saline. They spent more time in sleep after an initial hour of “quiet wake” after injection (Figure 3C).

To test whether CNO activates hM3Dq-expressing neurons *in vivo*, colocalization of c-Fos, a marker for neuron activity, with mCherry was examined after CNO or saline treatment. c-Fos was robustly expressed in MPB neurons after CNO administration, as compared to the saline control, indicating that hM3Dq effectively activated MPB neurons *in vivo* (Supplementary Figure 1). Moreover, c-Fos was highly expressed in many other nuclei such as the cerebral cortex, BF, thalamus, LH, TMN, ventral tegmental area (VTA), periaqueductal gray (PAG) and LC. This observation suggests that activation of MPB neurons strongly increased the activity of wake-promoting neurons.

### Optogenetic Activation of Glutamatergic MPB Neurons Immediately Initiated Wakefulness

**FIGURE 4:**
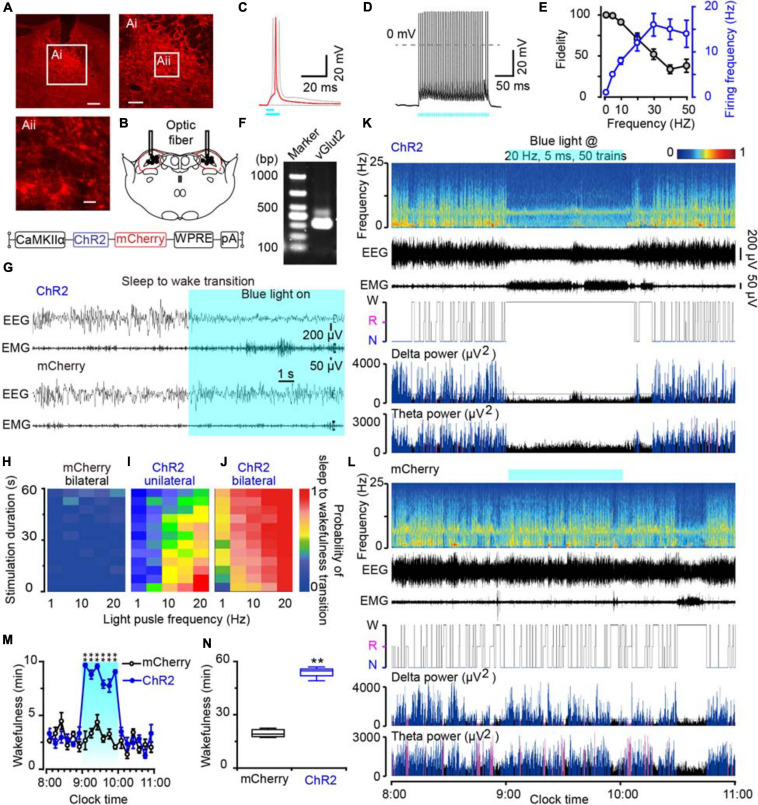
Photostimulation of MPB glutamatergic neurons induced a rapid wakefulness response. **(A)** Coronal brain section with native mCherry fluorescence confirmed ChR2-mCherry was confined in the MPB. Scale bars: A = 200 μm, Ai = 100 μm, Aii = 20 μm. **(B)** The schematic diagram showing the cannula trace and the optic fibers targeting the MPB. **(C)** A single action potential (red trace) evoked by 5 ms blue light pulses, and double action potentials (gray trace) evoked by >10 ms blue light pulses from a ChR2-expressing neuron. **(D)** Firing traces from a ChR2-expressing MPB neuron evoked by 10 Hz (5 ms pulses) photostimulation. **(E)** Neuronal firing showing an effective entrainment up to 20 Hz optogenetic stimulation of MPB neurons. **(F)** Single-cell RT-PCR analysis of ChR2-expressing neurons in MPB indicates the band of 315 bp represents VGluT2. **(G)** EEG/EMG examples of the sleep to wakefulness transition following optogenetic stimulation in a rat with bilateral ChR2 (top) or mCherry (bottom) expression in MPB glutamatergic neurons. **(H–J)** The probability of sleep to wakefulness transition in the frequency range of 1–20 Hz in mCherry controls **(H)**, stimulating the MPB unilaterally **(I)**, and bilaterally **(J)**. **(K,L)** Typical examples of EEG power spectra, EEG, EMG, hypnogram, and relative magnitude changes in delta and theta power in a rat injected with AAV-CaMKIIα-ChR2-mCherry **(K)** or AAV-CaMKIIα-mCherry **(L)** bilaterally. **(M,N)** The time course **(M)** and total amount **(N)** of wakefulness during optogenetic stimulation. **P < 0.01 compared with the mCherry groups (*n* = 6).

To clarify the role of the MPB neurons in initiating arousal, we employed the optogenetic strategy to activate glutamatergic MPB neurons using an AAV vector carrying an excitatory channelrhodopsin-2 (ChR2) with a CaMKIIα promoter [CaMKIIα-ChR2 (H134R)-mCherry-AAV, ChR2]. ChR2 was bilaterally expressed in MPB neurons (Figure 4A) and the optical fibers were placed within the MPB boundaries (Figure 4B). For the whole-cell current clamp recording conditions, short pulses of blue light (5–10 ms) elicited single spikes in MPB neurons (red curve, Figures 4C,D), whereas pulses longer than 10 ms induced two action potentials (light gray curve, Figure 4C). The trains of short blue light pulses entrained the firing of ChR2-expressing MPB neurons up to 50 Hz with high fidelity (*n* = 13, Figure 4E). We subsequently used a pulse duration of 5 ms and frequency of 20 Hz to stimulate the MPB glutamatergic neurons in the following optogenetic study *in vivo*. After patch clamp recordings, the cell type of the ChR2-expressing neurons, which can be elicited firing by light, was identified using single cell reverse-transcription PCR. The presence of a 315 bp-band specific for the vesicular glutamate transporter 2 (VGluT2, Figure 4F) suggests that firing was evoked in ChR2-expressing glutamatergic MPB neurons *in vitro* by blue-light stimulation.

Next, we implanted optic fibers and EEG electrodes on the rat skull with dental cement and placed EMG wire electrodes into the nuchal muscles. We then stimulated the glutamatergic MPB neurons *in vivo* with brief pulses (5 ms) of blue light in the frequency range of 1–20 Hz when the rats were sleep. Acute bilateral light stimulation produced immediate transitions from sleep to wakefulness with an average latency of 2.75 s in ChR2 rats at 20 Hz (Supplementary Movie 3). There was no striking change of the waveforms in mCherry-expressing control rats (*n* = 6, Figure 4G and Supplementary Movie 4). The probability of a sleep-to-wake transition increased with the stimulation frequency or bilateral stimulation in ChR2 rats (Figure 4J). Unilateral activation of MPB neurons also promoted transition from sleep to wakefulness with a lower probability than bilateral stimulation (Figure 4I), while there was no change in mCherry-expressing control rats (Figure 4H). Then chronic bilateral stimulation protocol (5 ms, 20 Hz, 50 trains, main interval 30 s) was applied to the MPB neurons *in vivo* for 1 h. As a result, activation of MPB neurons induced immediate transition from NREM sleep to wakefulness and the wakefulness was maintained for 1 h. After stopping stimulation, the wakefulness level of animals quickly returned to the baseline level (Figures 4K,M,N), while such effects were not observed in the MPB mCherry-expressing control rats (Figures 4L,M,N). These data show that the activation of glutamatergic MPB neurons resulted in immediate transitions from stable sleep to a continuous wakefulness.

### Activation of Glutamatergic MPB Neurons Controlled Wakefulness Through BF or LH Connections

**FIGURE 5:**
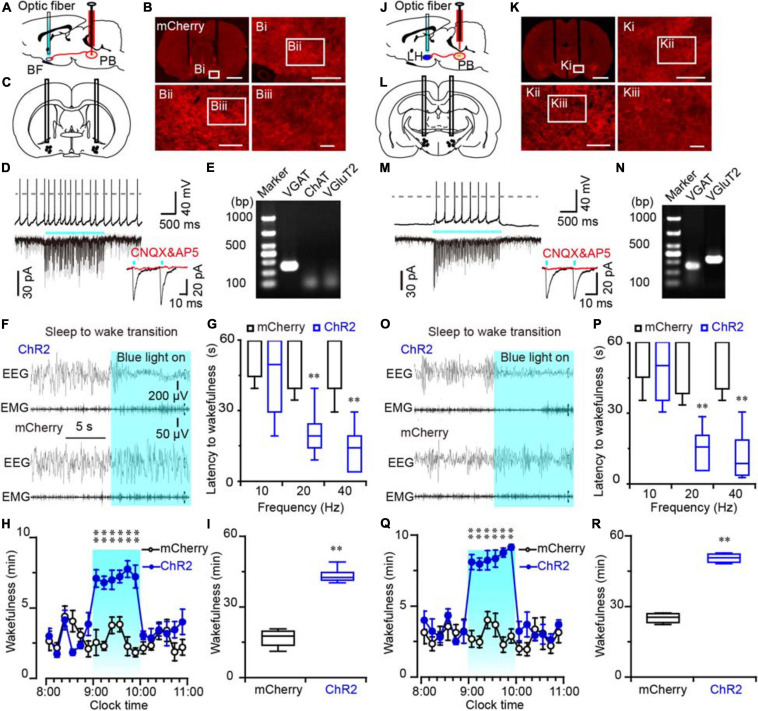
Photostimulation of ChR2-expressing MPB axonal terminals in the BF or LH evoked waking response in rats. **(A,J)** Diagram depicting the location of the optic fiber in the BF **(A)** or LH **(J)** of a rat injected with AAV-CaMKIIα-ChR2-mCherry or AAV-CaMKIIα-mCherry in the MPB. **(B,K)** Coronal sections with native mCherry fluorescence confirmed the expression of ChR2 protein in the BF **(B)** or LH region **(K)**. Scale bars: B, K = 2 mm; Bi, Ki = 500 μm; Bii, Kii = 200 μm; Biii, Kiii = 50 μm. **(C,L)** The schematic diagram showing the cannula traces and the optic fibers targeting the BF **(C)** or LH **(L)**. **(D,M)** Photostimulation of ChR2-expressing MPB axons increased firing frequency and evoked synchronized EPSCs in a BF **(D)** or LH neuron **(M)**; the evoked EPSCs were blocked by CNQX and AP5 (red trains). **(E,N)** Single-cell RT-PCR analysis of ChR2-expressing neurons in BF **(E)** and LH **(N)** indicates the bands represent VGluT2 or VGAT, respectively. **(F,O)** EEG/EMG examples of sleep to wakefulness transition following optogenetic stimulation in the BF **(F)** or LH **(O)** of a rat with bilateral ChR2 (top) or mCherry (bottom) expression in the MPB, respectively. **(G,P)** Latency of sleep to wakefulness after optogenetic stimulation in the frequency range of 10–40 Hz in BF **(G)** or LH **(P)**. **(H,I,Q,R)** The time course **(H,Q)** and total amount **(I,R)** of wakefulness during optogenetic stimulation in BF and LH, respectively. **P < 0.01 compared with mCherry groups (*n* = 6).

**FIGURE 6:**
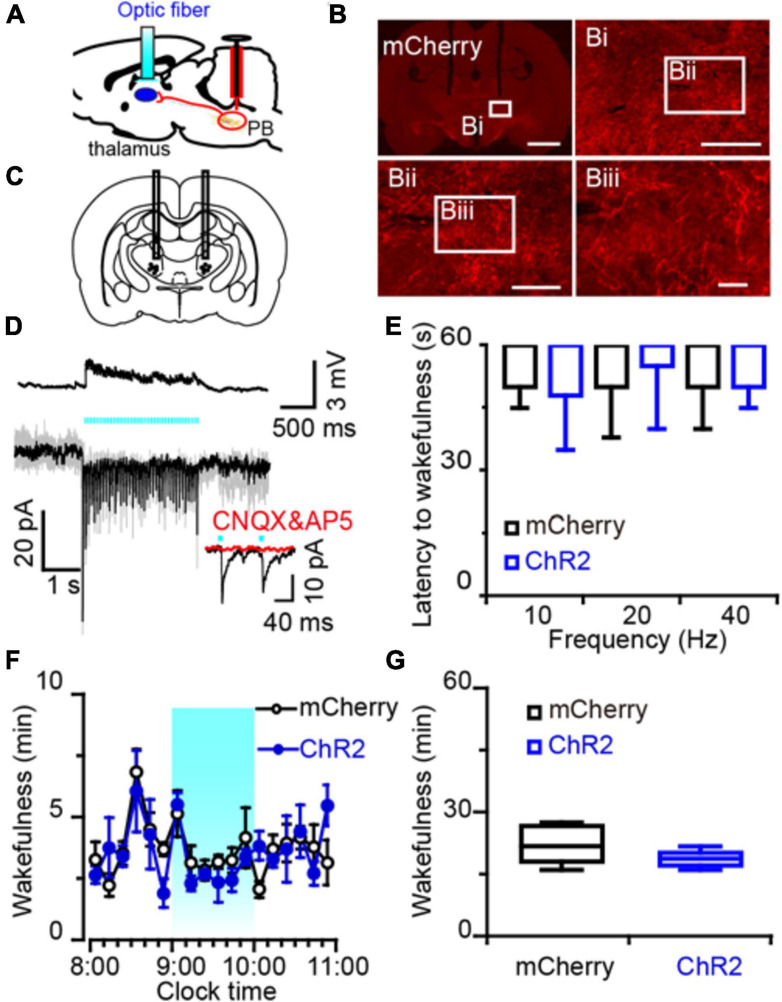
Photostimulation of MPB axonal terminals in the VM had no effect on wake response in rats. **(A)** Diagram depicting the location of the optic fiber in the VM of a rat injected with AAV-CaMKIIα-ChR2-mCherry or AAV-CaMKIIα-mCherry in the MPB. **(B)** Coronal sections with native mCherry fluorescence confirmed the expression of ChR2 protein in the VM. Scale bars: b = 2 mm; bi = 500 μm; bii = 200 μm; biii = 50 μm. **(C)** The schematic diagram showing the cannula traces and the optic fibers targeting the VM. **(D)** Optogenetic stimulation of MPB axons increased firing frequency and evoked synchronized EPSCs in VM neurons. Evoked EPSCs were blocked by CNQX and AP5 (red trains). **(E)** Latency of sleep to wakefulness after photostimulation in the frequency range of 10–40 Hz. **(F,G)** The time course **(F)** and total amount **(G)** of wakefulness during optogenetic stimulation in the LH, compared with mCherry groups (*n* = 6).

Although mapping of the c-Fos/mCherry expression may indicate a possible neuronal pathway for the promotion of wakefulness by the MPB, it is still unclear whether the robust expression of c-Fos is a direct effect of MPB activation or a secondary effect caused by consolidated arousal. Due to the low temporal resolution of c-Fos mapping, it is impossible to determine a causal link between nuclei activation and wakefulness. To demonstrate the neuronal circuits mediating the arousal promoting effect of the MPB, we expressed ChR2 in MPB (Supplementary Figure 2) and stimulated the ChR2-expressing glutamatergic MPB axons in the BF (Figures 5A–C), LH (Figures 5J–L), or VM (Figures 6A–C).

An increased firing frequency and excitatory post-synaptic current (EPSC) were elicited by blue light pulses in BF (Figure 5D), LH (Figure 5M), and VM neurons (Figure 6D). The signals evoked by stimulation of MPB neuron terminals were blocked by CNQX (6-cyano-7-nitroquinoxaline-2,3-dione, a competitive AMPA/kainate receptor antagonist) and AP5 [(2R)-amino-5-phosphonovaleric acid, a selective NMDA receptor antagonist], demonstrating that MPB-evoked signals are glutamatergic (Figures 5D,M), which is consistent with the single cell PCR results (Figure 4F). After patch clamp recordings, single-cell RT-PCR analysis revealed that all BF neurons that received excitatory afferent signals from MPB neurons were positive for the vesicular GABA transporter (VGAT, 250 bp) (6/6), but negative for ChAT (7/7) or VGluT2 (9/9) (Figure 5E). By contrast, glutamatergic signals from the MPB excited VGAT (8/8) or VGluT2 (7/7) positive neurons in the LH (Figure 5N). These results indicate that glutamatergic MPB neurons projected onto BF GABAergic neurons and LH GABAergic or glutamatergic neurons.

Next, EEG/EMG recordings together with optogenetic stimulation MPB terminals in the BF, LH, or VM were performed *in vivo* to reveal the neuronal circuits of the MPB in controlling wakefulness. Sleep-to-wake transition were decreased after blue light pulses at 5 ms in the frequency range of 20–40 Hz were bilaterally applied in the BF (Figures 5F,G) and LH (Figures 5O,P), but not in the VM (Figure 6E). To determine the effects of chronic stimulation on the wake-promoting effect of MPB axons, a long-term photostimulation protocol (5 ms, 40 Hz, 25 trains, main interval 30 s) for 1 h with sleep-wake recording were performed from 9:00 to 10:00 (when the sleep pressure is high in rats). Compared to control rats, bilateral optogenetic stimulation of MPB axons in the BF (Figures 5H,I) or LH (Figures 5Q,R) increased waking time by 2.59- and 2.87-fold, respectively, whereas wakefulness was not affected by stimulation of MPB axons in the VM (Figures 6F,G). These results provide fundamental evidence that glutamatergic MPB neurons excited BF GABAergic neurons or LH GABAergic and glutamatergic neurons, but not the VM neurons to initiate wakefulness.

### Chemogenetic Inhibition of PB Neurons in Rats Decreased Arousal

**FIGURE 7:**
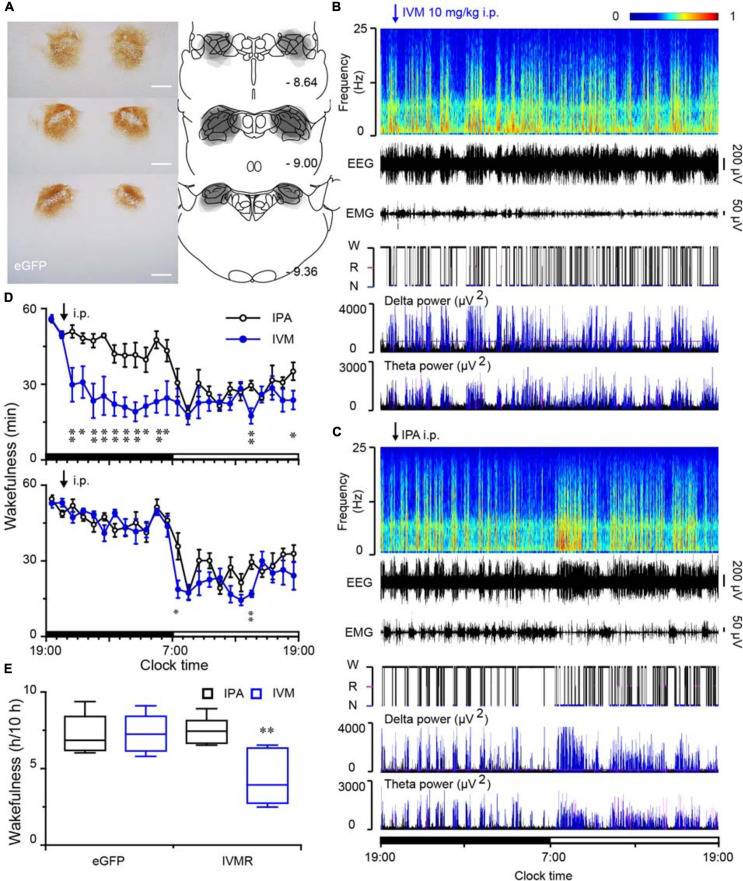
Chemogenetic inhibition of PB neurons decreased wakefulness in rats during the active period. **(A)** Brain section stained against eGFP to confirm IVMR expression in the PB. Injection sites were mapped on coronal atlas drawings at three different levels containing the PB. Superimposed eGFP expression areas in the PB of 6 AAV-injected rats are shown in the right panels. Scale bars: 1 mm. **(B,C)** Typical examples of EEG power spectra, EEG, EMG, hypnogram, and relative magnitude changes in delta and theta power over 24 h for the same rat given IVM **(B)** or IPA **(C)**. W, wakefulness; R, REM sleep; N, NREM sleep. **(D)** Time courses of wakefulness in rats after the administration of IVM (10 mg/kg) or IPA in IVMR rats and eGFP controls, respectively. **(E)** Amount of wakefulness during 10 h after IVM or IPA injection in IVMR and eGFP rats. *P < 0.05, **P < 0.01, compared with the IPA treatment (*n* = 6).

To explore the effect of inhibition of PB neurons in sleep-wake behavior, we employed an inhibitory chemogenetic tool, known as IVMR. IVMR are based on inhibitory human α1 glycine receptor with the mutations F207A and A288G to remove glycine sensitivity while producing IVM sensitivity (Lynagh and Lynch, 2010; Hu et al., 2014), and able to reduce neuronal excitability by mediating the influx of Cl^–^ (Hu et al., 2014; Obenhaus et al., 2016). AAV carrying IVMR (hSyn-IVMR-eGFP-AAV, IVMR) or eGFP (hSyn-eGFP-AAV, eGFP) was bilaterally injected into the PB. Immunohistochemistry against GFP revealed that IVMR fused to eGFP was successfully expressed in PB neurons, as indicated by superimposed eGFP expression areas in the PB of 6 AAV-injected rats (Figure 7A). During the dark period (when the rats are usually very active), i.p. administration of IVM (10 mg/kg) into rats expressing inhibitory IVMR in the PB neurons decreased the amount of wakefulness, as EEG delta amplitude was increased while the EMG amplitude decreased (Figure 7B). The vehicle treatment (isopropanol, IPA) did not significantly alter the sleep-wake profiles (Figure 7C). The inactivation of PB neurons bilaterally by IVM strongly decreased wakefulness for 10 h during the active period, as compared to the IPA control (Figure 7D). The total amount of wakefulness during 10 h decreased to 58% of the wake amount after IPA administration. By contrast, IVM did not change sleep-wake behavior in the eGFP control rats (Figures 7D,E). These data clearly indicated that PB neurons are essential for the maintenance of wakefulness under baseline conditions in rats.

## Discussion

In the current study, we employed chemogenetics to manipulate the activity of PB neurons, and found that activation of the MPB, but not the LPB by hM3Dq promotes wakefulness for 10 h, whereas inhibition of PB by IVMR resulted in a decrease in wakefulness for 10 h, indicating that MPB neurons are essential in controlling wakefulness in rats.

A pioneer clinical study used MRI scanning and postmortem histological analysis showed that brainstem stroke patients with coma have lesions or damage in the bilateral pontine tegmentum including PB, DR, and LC (Parvizi and Damasio, 2003). Later, chemical lesion of the entire PB and adjacent nuclei by orexin-saporin caused a behavioral unresponsiveness state with low-frequency cortical EEG in rats (Fuller et al., 2011), indicating the potential role of the pons in regulating wakeful consciousness. Moreover, chemogenetic activation of the PB induced long-lasting wakefulness during the light period when rats are normally mostly asleep (Qiu et al., 2016). Here we confirm and extend the findings that activation of the MPB is sufficient to induce robust continuous wakefulness for up to 10 h (Figure 1E). In addition, reversible chemogenetic inhibition of PB neurons by IVMR decreases wakefulness for 10 h during the dark period (Figure 7D), when rats are normally very active showing foraging, feeding, or exploring behaviors.

PB can be divided along the superior cerebellar peduncle into three main parts: MPB, LPB, and Kolliker-Fuse nucleus (KF) (Fulwiler and Saper, 1984; Singh et al., 2019). Several pioneer studies have revealed that LPB plays an important role in transmitting viscero- and somatosensory information to the forebrain, including pain, feeding and thermoregulation (Morrison and Nakamura, 2011; Le May et al., 2021), and the KF mainly involved in the respiration regulation (Chamberlin, 2004; Yang et al., 2020). To investigate the effect of MPB and LPB in sleep-wake regulation, hM3Dq were delivered into the MPB and LPB by stereotactic injection. We found that chemogenetic activation of MPB neurons induced continuous wakefulness, whereas activation of the LPB did not promote wakefulness in rats under basal conditions. To avoid non-specific expression of hM3Dq in the adjacent area of the targeted nucleus, the injection volume of the AAV was adjusted and confirmed by the immunostaining against the mCherry. The injection sites were centered in the MPB or LPB. In some rats, a small number of mCherry-containing neurons were observed in adjacent regions, such as the precoeruleus area (Figures 1A,B), raising the question of whether activation of the adjacent regions of MPB may induce wakefulness? However, from the previous lesion studies, lesions of the LC (Lu et al., 2006; Blanco-Centurion et al., 2007), PPT and LDT (Lu et al., 2006) have had no significant effect on wakefulness in rats. Only the lesion involved in the MPB region resulted in the dramatic decrease of wakefulness in rats (Fuller et al., 2011). Together with these pioneer studies, our results indicated the MPB is an essential nucleus in controlling wakefulness.

Kaur et al. (2013, 2017) reported that the specific lesion of glutamatergic LPB neurons did not alter the normal amounts of wakefulness or the EEG power spectrum. While blocking LPB signals decreased EEG arousal in response to hypercapnia, indicating that LPB neurons are important in hypercapnia-induced wakefulness, but not in controlling natural arousal (Kaur and Saper, 2019). Electrical activation of the glutamatergic LPB also induced reanimation (active emergence) during continuous isoflurane anesthesia, with a behavioral arousal and a significant decrease in EEG delta power in mice (Muindi et al., 2016). These results indicated that the LPB may regulate wakefulness secondary to the processing of viscero- and somatosensory information.

In contrast, specific deletions of glutamatergic MPB neurons decreased spontaneous wakefulness accompanied by an increase in both amount and EEG delta power of NREM sleep in mice (Kaur et al., 2013), and the population firing of MPB neurons was inhibited during sevoflurane-induced loss of consciousness (Xu et al., 2020), indicating the glutamatergic MPB neurons play an important role in controlling wakefulness. Moreover, chemogenetic inactivation of PB decreased wakefulness during the dark period (Figure 7), further indicating that the MPB is essential for controlling natural wakefulness. As previous reports have demonstrated that PB neurons are almost exclusively glutamatergic neurons (Liu and Jones, 1996; Yokota et al., 2007; Niu et al., 2010), some of which also express the calcitonin gene-related peptide, mu opioid receptors, or the corticotropin-releasing factor (Palmiter, 2018). The diversity of cell types in the MPB and LPB may lead to varying physiological functions. Although previous studies showed the projections of the MPB in rats are similar to those of the LPB (Saper and Loewy, 1980), how PB neurons innervate particular cell types in the targeted nuclei is still unclear. Further experiments are needed to identify the specific neural circuits of MPB or LPB for a specific behavior. Therefore, we consider that the MPB neurons are important for spontaneous wakefulness, while a subpopulation of LPB neurons may regulate arousal caused by visceral sensory distress, such as pain, extreme temperatures or respiratory insufficiency in rats.

Video analysis showed that the hM3Dq rats that received CNO injections are alert but are minimally moving around. The typical observed behavior, termed as attentive wake, is the movement of the head up and down and sideways without moving, accompanied by an increased theta EEG in the frequency range of 5–6 Hz (Valle et al., 1992; Lepski et al., 2012). During long-lasting wakefulness induced by MPB activation, rats may wish to gain a sense of visual depth, because PB is involved in visceral sensory regulation, such as taste, body temperature or anxiety (Baird et al., 2001; Bourgeais et al., 2001). By contrast, saline-treated hM3Dq rats or CNO-treated mCherry-expressing control rats spent more time in quiet wake (without head bobbing) with minimal grooming, exploring or feeding behavior during the first hour after the i.p. injection. In conclusion, our data provides fundamental evidence for the essential role of MPB in controlling wakefulness.

Previous studies proposed that possible neuronal PB circuits for promoting wakefulness are PB-BF/POA and the PB-LH (Anaclet et al., 2014; Qiu et al., 2016). Double staining against mCherry/c-Fos after activation of MPB neurons revealed that the expression of c-Fos increased remarkably in many regions including wake promoting nuclei such as the BF, thalamus, LH, VTA, and LC. The c-Fos expression pattern may indicated possible neuronal pathways mediating the wake promoting effect of MPB; however, c-Fos expression does not provide evidence for a causal relation between the observed neural activity and the ability to induce wakefulness.

Qiu et al. (2016) combined a retrograde strategy by injection of AAV6-Cre into the POA-BF, LH, or thalamic nucleus and the injection of AAV-DIO-hM3Dq into the PB area to activate specific neuronal circuits for sleep wake regulation. They found that activation of PB-BF or PB-LH pathway increased wakefulness for 4–5 h, which is much less than that induced by direct activation of PB somata. The reason for the short wakefulness duration may be due to the hM3Dq dense expressed in the LPB and spare in the MPB, which is in line with our finding that the LPB did not regulate the spontaneous wakefulness (Figure 1F). In addition, due to the injection of AAV6-Cre into huge areas of BF or PH, it is difficult to retrogradely label the exact target and the cell types in the MPB areas. Moreover, retrograde activation of the PB-LH/PH pathway may activate the PB-POA-BF pathway, or vice versa. Optogenetic methods used here to target the MPB terminals are much more accurate since they target the fibers issued from MPB neurons and are therefore more specific than retrograde-cre recombined floxed hM3Dq in which they target neurons projecting to the structures which could have collaterals to other structures.

To specific target the glutamatergic neurons in MPB, the excitatory ChR2 with a CaMKIIα promoter was employed and later confirmed by patch clamp recording and single cell PCR for the mCherry positive neurons (Figures 4, 5). Here, manipulation of the glutamatergic MPB neurons using optogenetics with high timing precision enabled us to analyze causality between neural activity and initiation and maintenance of wakefulness (Zhang et al., 2007). We found that acute photostimulation of glutamatergic MPB neurons expressing ChR2 immediately initiated and maintained wakefulness for 1 h during stimulation. Then optogenetic activation of MPB terminals revealed the neuronal circuits for controlling wakefulness mediated by GABAergic neurons in the BF and GABAergic and glutamatergic neurons in the LH.

The BF has been reported to contain glutamatergic, GABAergic and cholinergic neurons which regulate sleep-wake behaviors (Anaclet et al., 2015; Xu et al., 2015; Chen et al., 2016). The GABAergic neurons in the BF showed fast firing during wakefulness and REM sleep, tested by *in vivo* juxtacellular recordings (Hassani et al., 2009b). Furthermore, a pioneer electron microscopy results revealed that the GABAergic neurons in BF preferentially target cortical interneurons (Freund and Meskenaite, 1992). Later, a study using transgenic mice demonstrated that BF GABAergic projection neurons share many similarities with cortical interneurons, such as the fast firing, brief spikes and electrical coupling (McKenna et al., 2013). In addition, the cholinergic and glutamatergic neurons in the BF are also more active during wakefulness and REM sleep than during NREM sleep. Furthermore, activation of cholinergic, glutamatergic or parvalbumin-positive GABAergic neurons rapidly induces wakefulness (Xu et al., 2015), while inhibition of BF cholinergic neurons increased EEG delta power spectrum and decreased wakefulness (Anaclet et al., 2015; Chen et al., 2016). Here, we found that the glutamatergic MPB neurons excited the GABAergic BF neurons to initiate and maintain wakefulness, agreed with previous literature.

We demonstrated that the glutamatergic MPB neurons excite the GABAergic and/or glutamatergic neurons in the LH to control wakefulness. The LH contains several types of neurons critically implicated in the wake-sleep regulation, including glutamatergic, GABAergic, orexinergic and melanin-concentrating hormone containing neurons (Lin, 2000; Hara et al., 2001; Gerashchenko and Shiromani, 2004; Hassani et al., 2009a). Alam and Mallick (2008) reported that activation of glutamatergic neurons in and around the LH promoted arousal and suppressed both NREM and REM sleep, indicating that glutamatergic neurons of the LH play an important role in maintaining wakefulness. Additionally, optogenetic activation of LH GABAergic neurons exerts a strong wake-promoting effect in mice (Herrera et al., 2016), and similar effect was observed with chemogenetic activation of GABAergic LH neurons (Venner et al., 2016). In line with these findings, we postulate that glutamatergic MPB neurons innervate GABAergic and/or glutamatergic LH neurons to control wakefulness (Figure 5). However, whether there are other cell types in the LH mediated the wake promoting effect of glutamatergic MPB neurons still remained to be answered.

The thalamus is a large mass of gray matter and can be divided into many distinct portions, including the mediodorsal, paracentral, ventral lateral, submedius, VM, and so on. The thalamus participates in regulating many physiologic functions, such as relay of sensory and motor signals to the cerebral cortex, regulation of consciousness, and mediation of sleep and alertness (Kinomura et al., 1996; Maquet et al., 1996). PB neurons projected densely to midline and intralaminar thalamic nuclei of the rat (Herkenham, 1979; Krout and Loewy, 2000). We found that the increased c-Fos expression in the intralaminar, mediodorsal, lateral posterior and VM thalamic nuclei, after activation of glutamatergic MPB. However, a previous study showed lesion of the thalamic nuclei (Fuller et al., 2011), or activation of the PB-midline and intralaminar thalamus pathway did not alter sleep-wake behaviors significantly (Qiu et al., 2016). While the activities of matrix cells in the VM is high during wakefulness and low in NREM sleep, optogenetic activation of VM cells induced rapidly transitions from NREM sleep to arousal and chemogenetic inhibition of VM matrix cells decreased wakefulness (Honjoh et al., 2018), suggesting the VM plays a role in promoting arousal. Here, we clarified the functional connections between glutamatergic MPB and the VM by patch clamp recording, and demonstrated that this neural circuit is unexpected to be responsible for the wake-promoting effects of the MPB (Figure 6), although there are studies showing the VM participates in the catalepsy (Ossowska et al., 1986; Wullner et al., 1987).

In conclusion, we demonstrated the glutamatergic MPB neurons are essential in controlling wakefulness, and the wake-promoting effect of MPB mediated by BF GABAergic neurons and LH GABAergic or glutamatergic neurons in rats.

## Data Availability Statement

The original contributions generated for this study are included in the article/Supplementary Material, further inquiries can be directed to the corresponding authors.

## Ethics Statement

The animal study was reviewed and approved by Animal Care and Use Committee of Fudan University.

## Author Contributions

Z-LH, W-MQ, JL, and QX conceived and designed the study. QX, D-RW, HD, and LC carried out the experiments. QX, D-RW, Z-LH, and W-MQ analyzed the data. YC and ML provided the AAVs. QX, G-HC, W-MQ, and Z-LH wrote the manuscript. All authors contributed to the study and approved the final version.

## Conflict of Interest

The authors declare that the research was conducted in the absence of any commercial or financial relationships that could be construed as a potential conflict of interest.

## Supplementary Material

Supplementary Figure 1 Mapping of c-Fos expression in the rat brain after chemogenetic activation of MPB neurons. **(A–I)** Representative photomicrographs of c-Fos (black color) and mCherry (brown color) immunostaining in the rat brain. **(J)** The number of c-Fos-immunoreactive neurons in the rat brain after CNO or saline treatment. ^∗∗^*P* < 0.01 compared to Saline, assessed by student’s *t*-test (*n* = 4). MPB, medial parabrachial nucleus; CG, cingulate cortex; M1, primary motor cortex; S1, primary somatosensory cortex; LS, lateral septal nucleus; BNST, bed nucleus of stria terminalis; CeA, central amygdaloid nucleus; BF, basal forebrain; MDM, mediodorsal thalamic nucleus; LPMR, lateral posterior thalamic nucleus; PV, paraventricular thalamic nucleus; VM, ventromedial thalamic nucleus; LH, lateral hypothalamus; PSTN, parasubthalamic nucleus; TMN, tuberomammillary nucleus; MGV, medial geniculate nucleus; PAG, periaqueductal gray; VTA, ventral tegmental area; LC, locus coeruleus. Scale bars: CNO left panel, Saline left panel = 500 μm; CNO right panel, Saline right panel = 100 μm.

Supplementary Figure 2 Bilateral AAV injection sites in the rats MPB region. **(A)** Typical coronal brain sections of native mCherry fluorescence confirmed that the ChR2 protein was expressed in the MPB area at three brainstem levels. **(B–D)** Superimposed mCherry expression areas in the MPB of 6 AAV-injected rats are shown in each panel for the optogenetic stimulation of MPB axons in the BF **(B)**, LH **(C)**, and VM **(D)**. Scale bars: 1 mm.

Supplementary Table 1 The primers for single-cell RT-PCR.

Supplementary Movie 1 A MPB-hM3Dq rat whose behaviors were captured after the CNO injection. EEG and EMG recordings, and a video show that the rat spent most of its time in “attentive wake” characterized by head bobbing without moving around during the activation of MPB neurons by hM3Dq.

Supplementary Movie 2 A MPB-hM3Dq rat whose behaviors were captured after the saline injection. EEG and EMG recordings, and a video show that the rat spent more time in quiet wake, and sleep, and little time in grooming, exploring, eating and drinking during the first hour, followed by more sleep during the subsequent hours after saline treatment.

Supplementary Movie 3 A rat whose wakefulness was induced by blue light illumination of glutamatergic MPB neurons expressing ChR2. EEG and EMG recordings, and a video show that the rat was sleeping for at least 1 min before 1-min light illumination, which quickly produced wakefulness. The duration of the behavioral states of wakefulness and sleep are indicated above the EEG trace.

Supplementary Movie 4 A rat whose wakefulness was not induced by blue light illumination of glutamatergic MPB neurons expressing only mCherry. EEG and EMG recordings, and a video show that the rat was sleeping during the 4.5-min recording period, including the 1-min illumination period.
