# Supplementary figures and images for: Medial Parabrachial Nucleus Is Essential in Controlling Wakefulness in Rats
Source: Front Neurosci. 2021 Mar 25;15:645877. doi: 10.3389/fnins.2021.645877 (PMC8027131; doi:10.3389/fnins.2021.645877)

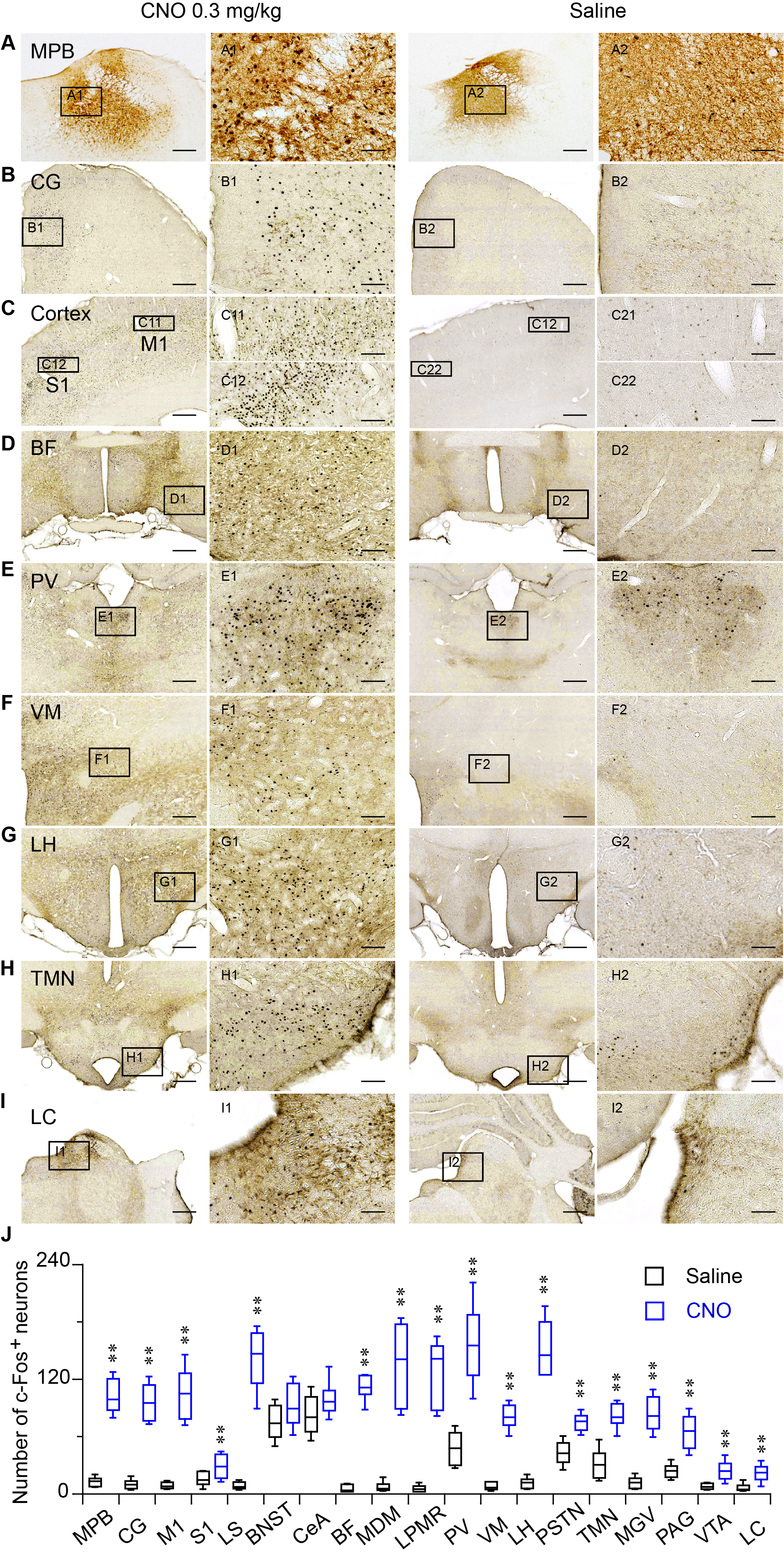

Supplement: Supplementary Figure 1 — Mapping of c-Fos expression in the rat brain after chemogenetic activation of MPB neurons. (A–I) Representative photomicrographs of c-Fos (black color) and mCherry (brown color) immunostaining in the rat brain. (J) The number of c-Fos-immunoreactive neurons in the rat brain after CNO or saline treatment. ∗∗P < 0.01 compared to Saline, assessed by student’s t-test (n = 4). MPB, medial parabrachial nucleus; CG, cingulate cortex; M1, primary motor cortex; S1, primary somatosensory cortex; LS, lateral septal nucleus; BNST, bed nucleus of stria terminalis; CeA, central amygdaloid nucleus; BF, basal forebrain; MDM, mediodorsal thalamic nucleus; LPMR, lateral posterior thalamic nucleus; PV, paraventricular thalamic nucleus; VM, ventromedial thalamic nucleus; LH, lateral hypothalamus; PSTN, parasubthalamic nucleus; TMN, tuberomammillary nucleus; MGV, medial geniculate nucleus; PAG, periaqueductal gray; VTA, ventral tegmental area; LC, locus coeruleus. Scale bars: CNO left panel, Saline left panel = 500 μm; CNO right panel, Saline right panel = 100 μm. [file Image_1.TIF]

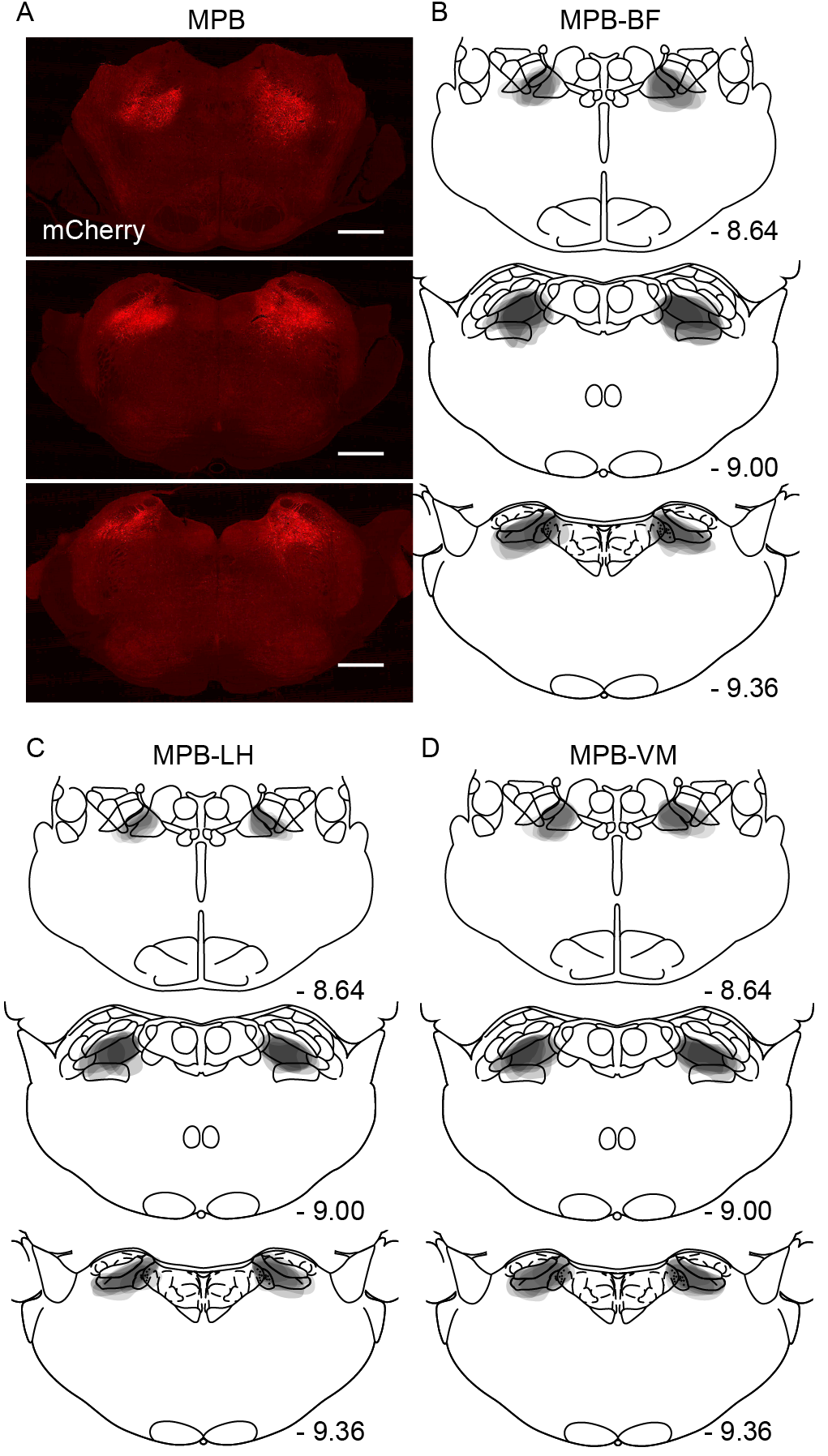

Supplement: Supplementary Figure 2 — Bilateral AAV injection sites in the rats MPB region. (A) Typical coronal brain sections of native mCherry fluorescence confirmed that the ChR2 protein was expressed in the MPB area at three brainstem levels. (B–D) Superimposed mCherry expression areas in the MPB of 6 AAV-injected rats are shown in each panel for the optogenetic stimulation of MPB axons in the BF (B), LH (C), and VM (D). Scale bars: 1 mm. [file Image_2.TIF]
