# Supplementary material for: Medial Parabrachial Nucleus Is Essential in Controlling Wakefulness in Rats
Source: Front Neurosci. 2021 Mar 25;15:645877. doi: 10.3389/fnins.2021.645877 (PMC8027131; doi:10.3389/fnins.2021.645877)
Supplement: Supplementary Table 1 — The primers for single-cell RT-PCR. [file Table_1.docx]

| **Supplementary Table 1. The primers for single-cell RT-PCR** | | | |
| --- | --- | --- | --- |
| Gene | primer pair | | Product size (bp) |
| VGluT2 | multiplex | Fwd: TGTTCTGGCTTCTGGTGTCTTACGAGAG | 600 |
|  |  | Rev: TTCCCGACAGCGTGCCAACA |  |
|  | nested | Fwd: AGGTACATAGAAGAGAGCATCGGGGAGA | 315 |
|  |  | Rev: CACTGTAGTTGTTGAAAGAATTTGCTTGCTC |  |
| VGAT | multiplex | Fwd: ATTCAGGGCATGTTCGTGCT | 650 |
|  |  | Rev: ATGTGTGTCCAGTTCATCAT |  |
|  | semi-nested | Rev: TGATCTGGGCCACATTGACC | 250 |
| ChAT |  | Fwd: ATGGCCATTGACAACCATCTTCTG | 324 |
|  |  | Rev: CCTTGAACTGCAGAGGTCTCTCAT |  |

Direction (5’ > 3’), VGluT2: vesicular glutamate transporter 2; VGAT: vesicular GABA transporters; ChAT: choline acetyl transferase.
